# Attenuation of NAD[P]H:quinone oxidoreductase 1 aggravates prostate cancer and tumor cell plasticity through enhanced TGFβ signaling

**DOI:** 10.1038/s42003-019-0720-z

**Published:** 2020-01-03

**Authors:** Dinesh Thapa, Shih-Bo Huang, Amanda R. Muñoz, Xiaoyu Yang, Roble G. Bedolla, Chia-Nung Hung, Chun-Liang Chen, Tim H.-M. Huang, Michael A. Liss, Robert L. Reddick, Hiroshi Miyamoto, Addanki P. Kumar, Rita Ghosh

**Affiliations:** 1 Department of Urology, School of Medicine, University of Texas Health Science Center at San Antonio, San Antonio, TX USA; 2 Department of Molecular Medicine, School of Medicine, University of Texas Health Science Center at San Antonio, San Antonio, TX USA; 3 Mays Cancer Center, School of Medicine, University of Texas Health Science Center at San Antonio, San Antonio, TX USA; 4 Department of Pathology, School of Medicine, University of Texas Health Science Center at San Antonio, San Antonio, TX USA; 5 Department of Pathology & Laboratory Medicine, University of Rochester Medical Center, Rochester, NY USA; 6 Department of Pharmacology, School of Medicine, University of Texas Health Science Center at San Antonio, San Antonio, TX USA; 7 South Texas Veterans Health Care System, San Antonio, TX USA; 8 Present Address: Department of Medical Oncology, Dana-Farber Cancer Institute, 450 Brookline Avenue, Boston, MA USA

**Keywords:** Prostate cancer, Cancer, Metastasis

## Abstract

NAD[P]H:quinone oxidoreductase 1 (NQO1) regulates cell fate decisions in response to stress. Oxidative stress supports cancer maintenance and progression. Previously we showed that knockdown of NQO1 (NQO1^low^) prostate cancer cells upregulate pro-inflammatory cytokines and survival under hormone-deprived conditions. Here, we tested the ability of NQO1^low^ cells to form tumors. We found NQO1^low^ cells form aggressive tumors compared with NQO1^high^ cells. Biopsy specimens and circulating tumor cells showed biochemical recurrent prostate cancer was associated with low NQO1. NQO1 silencing was sufficient to induce SMAD-mediated TGFβ signaling and mesenchymal markers. TGFβ treatment decreased NQO1 levels and induced molecular changes similar to NQO1 knockdown cells. Functionally, NQO1 depletion increased migration and sensitivity to oxidative stress. Collectively, this work reveals a possible new gatekeeper role for NQO1 in counteracting cellular plasticity in prostate cancer cells. Further, combining NQO1 with TGFβ signaling molecules may serve as a better signature to predict biochemical recurrence.

## Abstract (web summary)

Thapa et al find that depletion of the antioxidant enzyme NAD[P]H:Quinone Oxidoreductase 1 (NQO1) accelerates prostate tumorigenesis and induces the epithelial-to-mesenchymal transition by activating TGFβ signaling. They also find that low NQO1 is associated with mesenchymal signature and biochemical recurrence in clinical samples.

## Introduction

Cancer cells are under increased oxidative stress, presumably associated with uncontrolled growth, metastasis, and response to therapy. Cancer cells have an intrinsic antioxidant defense system through a battery of antioxidant enzymes to adapt to increased oxidative stress^1^. NAD[P]H:quinone oxidoreductase 1 (NQO1) is a key component of the antioxidant defense system. NQO1 is a cytosolic enzyme that detoxifies quinones and protects cells against oxidative stress. Several studies have revealed protective roles for NQO1 that apparently are unrelated to its enzymatic activities. For example, NQO1 directly binds to the tumor suppressor p53 protein and stabilizes it against proteasomal degradation ultimately making cell fate decisions in response to endogenous and exogenous stress^2,3^.

Oxidative stress is recognized as an important contributor to the transition of epithelial cells to mesenchymal phenotype (EMT), a reversible program that enables metastasis^4^. This switch in cell differentiation and behavior is mediated by key transcription factors, including SNAIL, zinc-finger E-box-binding (ZEB), and basic helix-loop-helix transcription factors^5^. EMT is characterized by upregulation of mesenchymal-associated genes, such as N-cadherin, vimentin and fibronectin, and decreased expression of epithelial-associated genes such as E-cadherin^5^. These changes in cell plasticity are associated with aggressive cell behavior including migration, invasion, tumor cell survival, stemness, resistance to radiation and chemotherapy in various cancer types including prostate cancer^6^. The reversible nature of the EMT process ensures that mesenchymal cells undergo differentiation back to epithelial phenotype (mesenchymal-epithelial transition; MET). Therefore, these transient molecular changes are initiated and controlled by signaling pathways that respond to extracellular cues such as oxidative stress and not by permanent alteration^5,7^. Transforming growth factor-β (TGFβ) family plays a leading role in this signaling process.

TGFβ is a pleotropic cytokine, which upon activation causes autocrine and paracrine effects on tumor cells and the tumor microenvironment, thus regulating a variety of cellular processes, including angiogenesis, apoptosis, migration, EMT, and metastasis^8,9^. Several studies have suggested that in early-stage cancers, TGFβ signaling may be growth suppressive, while in advanced stages, TGFβ promotes invasion and metastasis^10,11^. TGFβ signaling in prostate cancer cells increases the expression of numerous genes associated with the development of bone metastases and TGFβ inhibition effectively reduces prostate cancer bone metastases^12^. Recent studies highlight the immunosuppressive role of TGFβ leading to immune evasion therefore it can be targeted to improve cancer immunotherapy^13,14^. TGFβ receptors initiate downstream signaling through either SMAD-mediated canonical signaling or SMAD-independent non-canonical signaling. The canonical signaling pathway involves phosphorylation of SMAD2 or SMAD3 with SMAD4 and nuclear translocation to regulate gene expression with the help of transcriptional coactivators or corepressors^11^.

Prostate cancer is one of the most commonly diagnosed malignancy accounting for almost 1 in 5 cancer diagnoses among men in the United States^15^. Oxidative stress, a hallmark of prostate cancer is correlated with tumor metastasis and therapeutic resistance in primary tumors^16–18^. We had reported that NQO1 attenuation fueled pro-inflammatory signaling and promoted androgen-independent prostate cancer cell survival^19^. Interestingly, Kurfurstova et al. observed a focal loss of NQO1 along with loss of PTEN in advanced prostate cancer lesions suggesting NQO1 may have a tumor suppressive role and its loss may facilitate tumor progression^20^. In contrast, recent reports suggest a pro-tumorigenic role of NQO1 in breast and colon cancers since high expression was implicated in disease progression and metastasis^21,22^. Despite these paradoxical but critical observations regarding NQO1 in various cancers, and the contribution of oxidative stress and inflammation to prostate cancer development and progression, the clinicopathological significance of NQO1 in prostate cancer and the molecular mechanisms underlying NQO1 regulation have not been fully elucidated. Our goal in this work was to understand how NQO1 contributes to prostate carcinogenesis^16,23^.

Here we show that indeed NQO1 inhibition enhanced prostate tumorigenesis in an orthotopic model. NQO1 message and protein is reduced in advanced prostate cancer, is associated with survival in multiple cohorts and biochemical recurrence (BCR) in circulating tumor cells (CTC) obtained from prostate cancer patients. We also show that suppression of NQO1 induced EMT through TGFβ signaling activation. TGFβ treatment of prostate cancer cells decreased NQO1 levels and mimicked molecular changes of NQO1 knockdown cells. Together our results provide evidence that NQO1 acts as a guardian to protect prostate cancer cells from undergoing TGFβ-mediated EMT changes that are associated with advanced disease progression.

## Results

### NQO1 knockdown accelerates prostate tumorigenesis

**Fig. 1:**
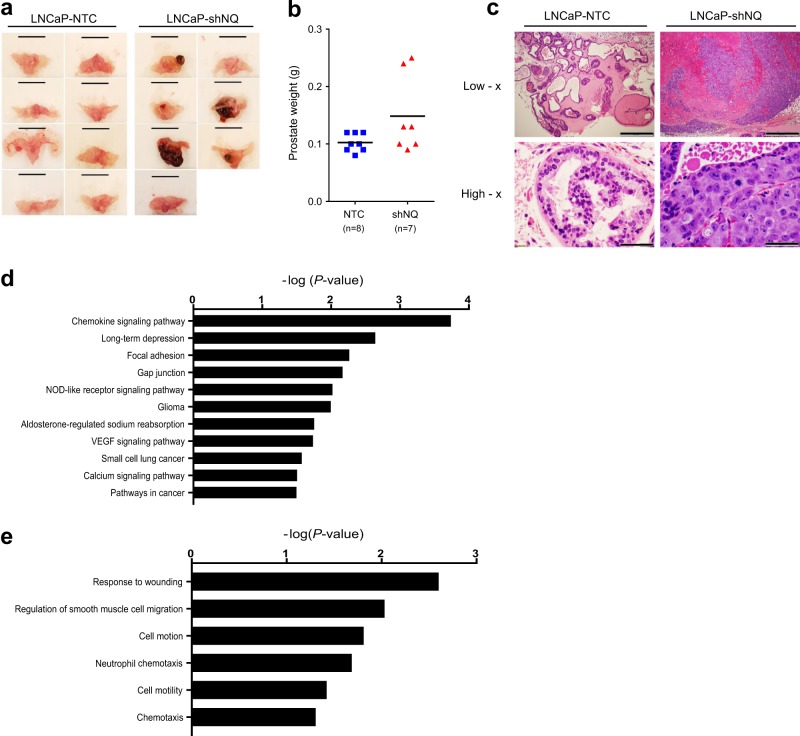
NQO1 knockdown enhances prostate tumorigenesis. **a** Prostate and seminal vesicles showing tumor burden in animals with orthotopic injection of NTC and shNQ cells. **b** Weights of prostate and prostate + tumor. **c** Representative images of H&E-stained sections from the prostate and tumor. Scale bars, 500 µm (low x) and 50 µm (high x). **d**, **e** Genome wide expression changes between NTC and shNQ cells were analyzed for pathways and functions using DAVID web tool. DAVID-generated modified Fisher’s exact test *p*-values of selected terms are represented in log-10 scale.

Our previous work showed that NQO1^low^ cells upregulated inflammatory signaling^19^. To determine the impact of low NQO1 on prostate tumorigenesis, stably silenced NQO1 (shNQO1) cells and non-target control (NTC) were implanted into the prostate of athymic nude mice, followed by analysis of tumor growth. At the time of euthanasia, five of seven animals with shNQO1 cells developed tumors, while there was no incidence of tumor in mice (0/8) implanted with NTC cells (Fig. 1a). Prostates from animals bearing shNQO1 tumors weighed more than those from animals implanted with NTC cells (Fig. 1b). Pathological analyses showed that five of seven animals in the shNQO1 group developed poorly differentiated carcinoma (PDC), while none of the animals in the NTC group developed PDC (Fig. 1c and Table 1). All animals in the NTC group developed high-grade intraepithelial neoplasia (HGPIN) which we categorized into 0–3 grades based on their histology (Supplementary Table 1). We then analyzed our microarray data set of shNQO1 and NTC cells (GSE58336)^19^. Using DAVID (Database for Annotation, Visualization and Integrated Discovery) web tool, we identified several pathways associated with tumorigenesis including functions related to wounding, chemotaxis, and migration (Fig. 1d, e). To our knowledge, this study shows for the first time that NQO1 knockdown enhances prostate tumor development in vivo.

**Table 1:** The number (%) of mice that developed high-grade prostatic intraepithelial neoplasia and poorly differentiated carcinomas.

Group	Gross advanced tumor	H&E pathology grading		
		Grade 2	Grade 3	PDC
	*n* (%)	*n* (%)	*n* (%)	*n* (%)
LNCaP-NTC	0/8 (0%)	8/8 (100%)	4/8 (50%)	0/8 (0%)
LNCaP-shNQ	5/7 (71%)	6/7 (86%)	5/7 (71%)	5/7 (71%)

*PDC* poorly differentiated carcinoma

### Reduced NQO1 is associated with advanced prostate cancer

**Fig. 2:**
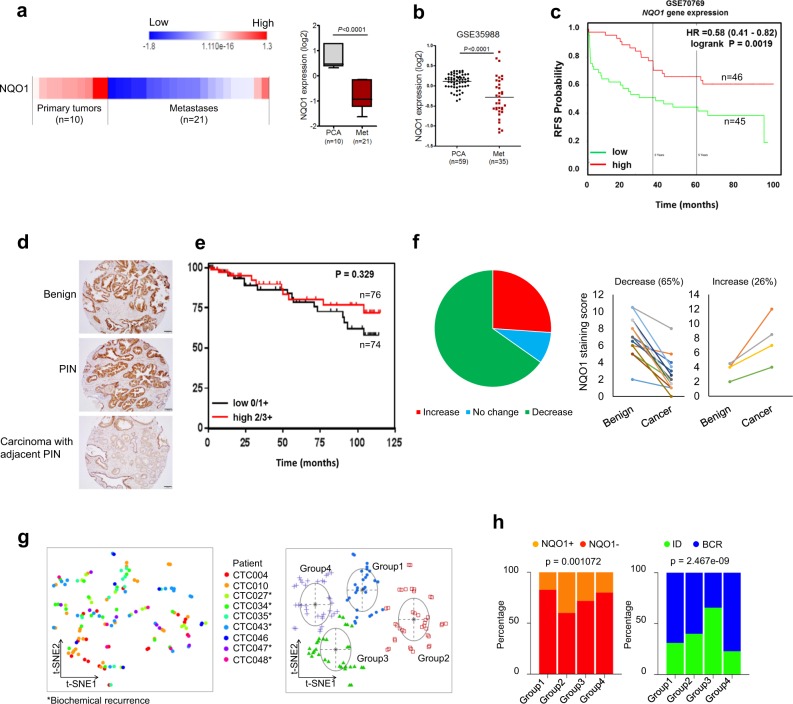
NQO1 is suppressed in advanced prostate cancer and metastatic tissues. **a** NQO1 expression in primary prostate tumors and in samples from distant metastases (Chandran dataset). Expression level is presented as boxplot (*P* < 0.0001 with unpaired *t*-test). **b** Scatter plot analysis of NQO1 expression in tumor and metastasis (GSE35988). **c** Kaplan–Meier analysis of recurrence-free survival (RFS) was compared between high and low NQO1 transcript levels using median gene expression value as a bifurcating point. RFS plot of 91 prostate cancer patients from GSE70769. **d** Representative photographs of immunohistochemical detection of NQO1 in a tumor microarray with a cohort representing benign, PIN and carcinoma lesions obtained at the time of prostatectomy (*n* = 150). Scale bars, 100 µm (**e**). Kaplan–Meier analysis of recurrence-free survival in the TMA (from above) based on low (0/1+) and high (2+/3+) immunostaining (*P* = 0.329 with log-rank analysis). **f** Composition of the 23 BCR patients from the TMA samples with a NQO1 change. Graphs showing cumulative IHC staining scores from benign and cancer sites from each patient. **g** t-Distributed Stochastic Neighbor Embedding (t-SNE) plot of unbiased clustering of 136 CTCs from patients (*n* = 9) based on *NQO*1 and TGF-β pathway scores, and identification of four groups in different colors. **h** NQO1 expressed as percentage in each group. Initial diagnosis (ID) and biochemical recurrence (BCR) ratio in different groups (p values calculated by Chi-square test).

Analysis of publicly available datasets for NQO1 expression in surgical specimens showed significantly lower expression in metastatic tumors (liver, lymph node, lung, adrenal; (*P* < 0.0001 with unpaired *t*-test) compared with primary prostate tumors (Fig. 2a)^24^. Expression data from GSE35988 showed significant association (*P* < 0.0001 with unpaired *t*-test) between low expression of NQO1 and metastatic prostate cancer^25^ (Fig. 2b). NQO1 expression decreased consistently in metastatic prostate cancer samples in multiple independent studies^26–30^ (Supplementary Fig. 1). To determine whether NQO1 predicts prostate cancer patient outcomes, we investigated the correlation and prognostic implication of NQO1 in two independent cohorts GSE70769 and GSE40272 using PROGgeneV2^31^. Consistent with the expression of low NQO1 and tumor metastasis in multiple cohorts, a strong association was observed between NQO1 mRNA expression and prognosis of prostate cancer patients. Patients with low NQO1 expression had poorer relapse free survival (Fig. 2c and Supplementary Fig. 2).

We then used a set of tissue microarray (TMA) consisting of a cohort of 150 radical prostatectomy specimens. NQO1 was detectable mainly in the cytoplasmic compartment of epithelial cells from benign, high-grade prostatic intraepithelial neoplasia (PIN), and infiltrating adenocarcinoma. In multiple cases, there was a notable decrease in NQO1 level (0 to 1+) in carcinoma compared with PIN from the same patient that showed strong staining (2+ to 3+) (Fig. 2d). Low levels (0 to 1+) of NQO1 were seen in 49% of carcinomas versus 30% and 37% of benign and PIN tissues, respectively (*P* < 0.05) (Supplementary Table 2). In particular, 22 (56%) of 39 advanced tumors (i.e., pT3-4 and/or pN1) showed low NQO1 expression. However, analyses of clinicopathological data of this cohort showed no statistically significant correlations between the level of NQO1 protein and Gleason score (GS), pathologic stage (pT), and lymph node metastasis (pN) (Supplementary Table 3). There was a trend of an association between low NQO1 protein level and decreased recurrence-free survival, however the difference was not significant (*P* = 0.329; Fig. 2e) in this small cohort.

Upon further analysis of the immunohistochemistry data from the TMA samples, we observed a link between decreased level of NQO1 in carcinoma compared with normal and biochemical recurrence (BCR) following radical prostatectomy (Fig. 2f). Out of a total of 26 biochemical recurrent (BCR) patients in this TMA cohort, 23 BCR patients were subjected to cumulative NQO1 staining analysis since normal prostate tissues were not available in three cases. Of these patients, 15 (65%)/6 (26%)/2 (9%) showed a decrease/ an increase/no change respectively in NQO1 score in carcinomas. In addition, 10 (67%) out of 15 patients with low levels of NQO1 had advanced tumor (pathological stage pT3-4) while 7 (70%) of 10 in high NQO1 group had organ confined tumor (pathological stage pT2). We also used single-cell RNA sequencing expression profiles of CTCs from patients who developed BCR (GSE115501). The transcriptional scores of NQO1 is low (NQO1^−^) in 73% (99/136) of CTCs (Supplementary Fig. 3a). Multidimensional scaling of these patients, based on their transcriptional scores (NQO1 and TGFβ signature; Supplementary Fig. 3b), identified four groups (Fig. 2g). Groups 1 and 4 with low NQO1 expression were significantly associated with BCR (Fig. 2h; *P* = 2.467e-09 with Chi-square test) suggesting NQO1 attenuation in BCR patients. These findings establish the clinical significance of NQO1 in advanced prostate cancer and underscore the need to investigate the impact of reduced NQO1 expression on prostate cancer progression.

### Low NQO1 is associated with mesenchymal attributes

**Fig. 3:**
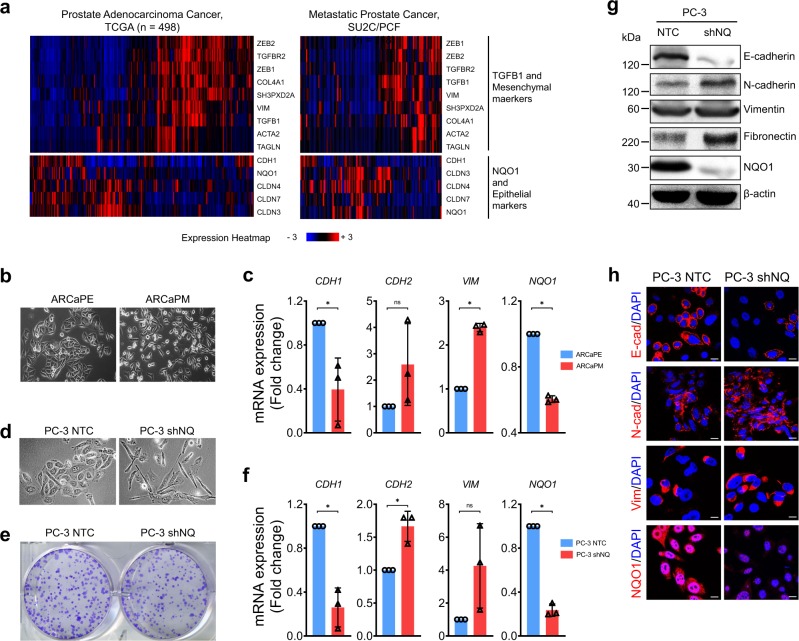
NQO1 expression correlates with epithelial markers. **a** Cluster heatmap of selected gene signature from two independent cohorts, TCGA and SU2C/PCF Dream Team was accessed and created using cBioPortal. **b** Representative microscope images of ARCaP_E_ and ARCaP_M_ cells showing typical morphological features associated with epithelial and mesenchymal phenotypes respectively. **c** Comparison of EMT marker gene expression (*CDH1*, *CDH2,* and *VIM*) and *NQO1* between ARCaP_E_ and ARCaP_M_ cells by qPCR analysis (*P < 0.05). **d** Representative images to show morphology PC-3 cells stably transduced with non-target control (NTC) and NQO1-specific (shNQ) shRNA. **e** Colony formation of PC-3 NTC and shNQ cells. **f** Changes in epithelial (*CDH1*) and mesenchymal (*CDH2* and *VIM*) markers following NQO1 KD in PC-3 cells. **g** Representative immunoblots of changes in EMT related markers in NTC and shNQ cells. β-actin is loading control. **h** Representative immunofluorescence images of changes in EMT markers in NTC and shNQ cells. All experiments were repeated thrice. Scale bars, 20 µm.

To understand the link between NQO1 and tumor cell plasticity, we analyzed publicly available datasets of two large clinical cohorts, TCGA (*n* = 498) and SU2C/PCF (*n* = 118). Cluster heatmap of metastatic transcriptional signature with TGFβ (*TGFB1*, *TGFBR2*), Fibroblast-TGFβ response (*ACTA2*, *COL4A1*, *TAGLN*, *SH3PXD2A*) and EMT (*CLDN3*, *CLDN7*, *CLDN4*, *CDH1*, *VIM*, *TWIST1*, *ZEB1*, *ZEB2*) along with *NQO1* were created. The correlation gene expression pattern showed that *NQO1* expression is consistently clustered with epithelial signature and inversely correlated with TGFβ activation and mesenchymal gene signature (Fig. 3a). We then tested whether NQO1 activity is suppressed as epithelial cells undergo transition to mesenchymal phenotype. The establishment of isogenic ARCaP_E_ (epithelial) and ARCaP_M_ (mesenchymal) cells from parental ARCaP cells by Xu et al.^32^ provided an important tool to characterize crucial players involved in EMT transition. Morphologically ARCaP_M_ cells have distinct mesenchymal characteristics including elongated appearance and dispersed cell–cell adhesion (Fig. 3b). As expected, these cells had decreased *CDH1* and increased *CDH2* and *VIM* expression (Fig. 3c) compared with ARCaP_E_ cells. Given our previous observations that NQO1 inhibition fueled migration and androgen-independent cell survival^19^, we examined the involvement of NQO1 in EMT. Indeed, we found that *NQO1* expression is significantly repressed in ARCaP_M_ cells (Fig. 3c; *P* < 0.05). Immunoblotting and immunofluorescence analyses validated the expression observations (Supplementary Fig. 4a, b). We used cell survival against β-lapachone (β-lap) as a read-out of NQO1 functional activity since β-lap selectively kills NQO1 expressing cancer cells by generating a futile redox cycle^33^. ARCaP_E_ cells were more sensitive to β-lap at both 2.5 and 5 µM doses while ARCaP_M_ cells were significantly less sensitive with about 30% less cells killed at equivalent doses. Furthermore, the NQO1-specific inhibitor MAC220 significantly inhibited β-lap-induced cell death in ARCaP_E_ cells (Supplementary Fig. 4c; *P* < 0.05 with unpaired *t*-test).

To determine whether NQO1 silencing is sufficient to induce EMT-like changes in prostate cancer cells, we used PC-3 shNQ cells because they were morphologically dispersed and more efficient in wound closure^19^. Indeed, NQO1 knockdown in PC-3 cells exhibited distinct morphological changes, including cell elongation and junctional disruption (Fig. 3d). PC-3 shNQ cells were slower in colony formation and the colonies formed were irregular and diffused (Fig. 3e). Consistent with these phenotypic changes, expression of *CDH1* was suppressed and that of *CDH2* and *VIM* increased (Fig. 3f). Immunoblotting and immunofluorescence showed a dramatic repression of E-cadherin, and concurrent upregulation of N-cadherin, vimentin and fibronectin protein levels in NQO1 inhibited cells (Fig. 3g, h). These observations together suggest a regulatory role for NQO1 during the transition of tumor cells from epithelial to mesenchymal phenotype.

### NQO1 inhibitors increase cell migration

**Fig. 4:**
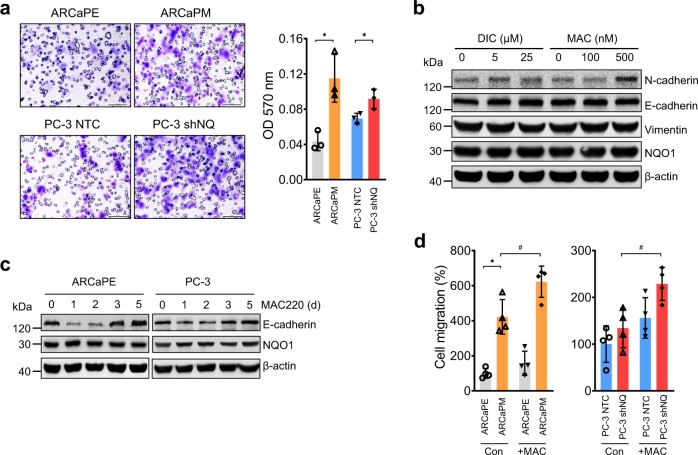
NQO1 inhibition increases cell migration. **a** Representative fields of crystal violet stained images of ARCaP and PC-3 cells that migrated through 8 μm transwell plates. Scale bars, 100 µm. The bar graph shows significantly increased cell migration in NQO1^low^ clones (ARCaP_M_ and PC-3 shNQ) (*p* < 0.05). mean ± SD of *n* = 3 independent experiments. **b** Immunoblotting to show changes in EMT markers following treatment with increasing doses of NQO1 inhibitors (Dicoumarol or MAC220). β-actin was loading control. **c** Time kinetics of changes in E-cadherin levels following treatment of ARCaP_E_ and PC-3 cells with MAC220 for 0–5 d. β-actin is the loading control. **d** Changes in migration of ARCaP_M_ and PC-3 shNQ cells upon inhibition of NQO1 activity with MAC220 treatment (# *p* < 0.05). mean ± SD of *n* = 4 independent experiments.

Analysis of cell migration by Transwell assay showed significantly increased migration in ARCaP_M_ and PC-3 shNQ cells compared respectively with ARCaP_E_ and PC-3 NTC cells (Fig. 4a; *P* < 0.05). Catalytic inhibition of NQO1 with Dicoumarol and MAC220 in PC-3 cells increased N-cadherin levels (Fig. 4b). Interrogation of the kinetics showed that MAC220 inhibited E-cadherin level for 2 days and was later restored (Fig. 4c). Restoration of E-cadherin suggests the plastic nature of these cells and may be due to the signals for constant cell rearrangement for cell–cell and matrix contact. To confirm and extend this finding, we performed cell migration assay with a catalytic NQO1 inhibitor, MAC220 that further augmented migration (*p* < 0.05) (Fig. 4d). Taken together, these results show that NQO1 inhibition plays a critical role in the repression of E-cadherin and enhancing the motility of prostate cancer cells.

### NQO1 influences essential components of TGFβ signaling

**Fig. 5:**
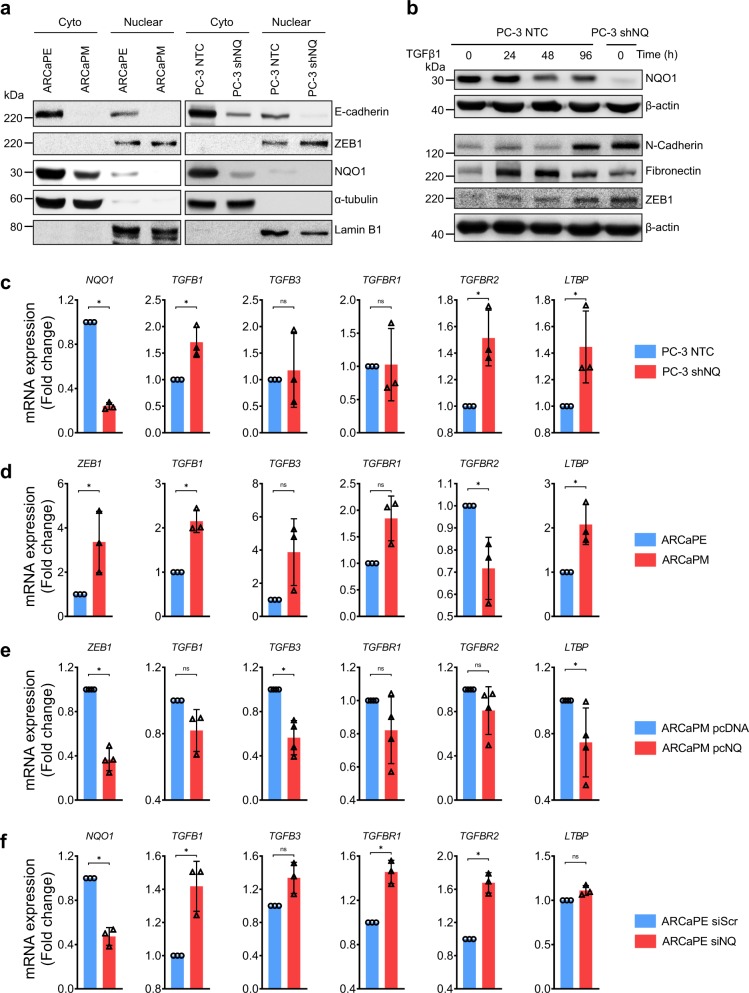
NQO1 regulates TGF-β signaling associated genes. Western blotting to show changes in EMT markers in (**a**) cytoplasmic and nuclear extracts from ARCaP and PC-3 cells (**b**) whole cell lysates from PC-3 cells. **c**, **d** mRNA expression changes in TGFβ-associated genes detected by qPCR between PC-3 NTC and PC-3 shNQ cells (**c**) and ARCaP_E_ and ARCaP_M_ cells (**d**). **e** Changes in mRNA expression of TGFβ-associated gene expression following overexpression of pcDNA NQO1 (pcNQ) in ARCaP_M_ cells. **f** Changes in mRNA expression of TGFβ-associated gene expression following transient knockdown of NQO1(siNQ) in ARCaP_E_ cells. All qPCR experiments were run in duplicate and repeated three to four times.

Several transcription factors including SNAI1 and ZEB1 cooperate to affect E-cadherin repression. We analyzed nuclear and cytoplasmic protein fractions and found increased nuclear ZEB1 in both NQO1^low^-ARCaP_M_ and PC-3 shNQ cells (Fig. 5a). Next, we investigated the possible involvement of TGFβ in PC-3 shNQ cells. We observed that the EMT-like changes observed in the morphology of PC-3 NTC cells stimulated with TGFβ1 were comparable to PC-3 shNQ cells (Supplementary Fig. 5). We found notable NQO1 inhibition by TGFβ1 stimulation (Fig. 5b). It is possible that TGFβ influences NRF2 signaling to decrease NQO1 levels^34^. NQO1-silencing mediated increase in N-cadherin, fibronectin, ZEB1 mimicked TGFβ1-stimulated effects on PC-3 NTC cells. Overall, NQO1 knockdown recapitulates the effect of TGFβ-activation in PC-3 cells (Fig. 5b). TGFβ-signaling was indeed activated in PC-3 shNQ cells as seen by increased expression of *TGFB1* and its receptor *TGFBR2*. Additionally, increased expression of the latent TGFβ binding protein (*LTBP*, that converts the latent form to active TGFβ state) was an indication of TGFβ signaling activation (Fig. 5c). Similarly, increased expression of genes associated with active TGFβ-signaling including *ZEB1, TGFB1*, *TGFB3*, *TGFBR1,* and *LTBP* was observed in ARCaP_M_ cells (Fig. 5d). To determine the protective role of NQO1 in EMT, we established ARCaP_M_ cells that stably overexpress NQO1 (Supplementary Fig. 6). Expression of NQO1 partially reversed the expression of TGFβ-associated genes observed in NQO1 ^low^ ARCaP_M_ cells (Fig. 5e). Conversely, siRNA-mediated inhibition of NQO1 in NQO1^high^, ARCaPE cells significantly increased TGFβ and its receptors even at 50% inhibition of NQO1 (Fig. 5f; *P* < 0.05). These results together suggest that NQO1 influences plasticity and migration of prostate cancer cells by restraining TGFβ signaling.

### NQO1 knockdown mimics TGFβ signaling activation

**Fig. 6:**
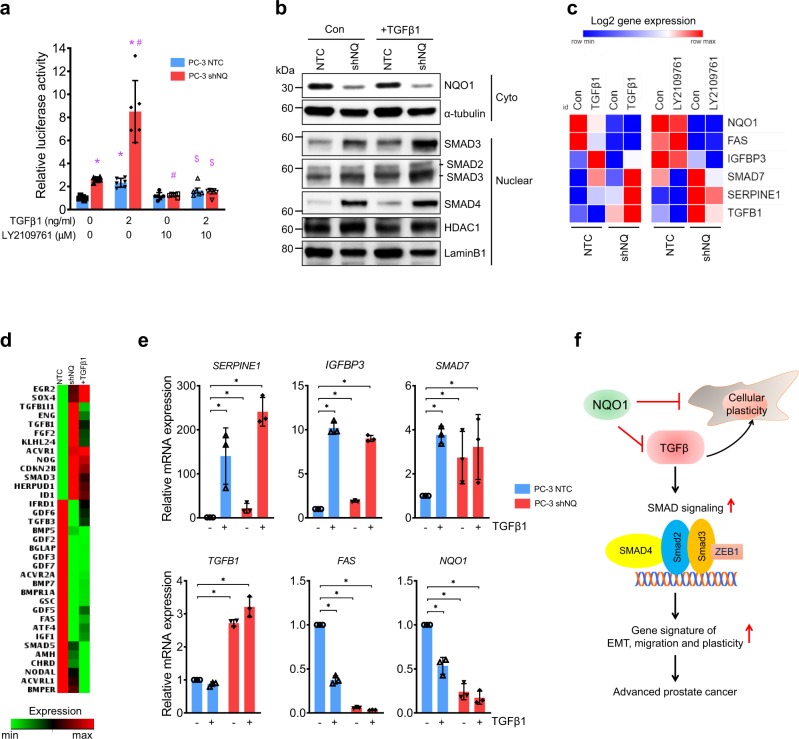
Activation of TGFβ signaling in NQO1 knockdown cells. **a** SMAD3 and SMAD4 reporter luciferase activity in PC-3 NTC and PC-3 shNQ cells transiently transfected with SBE4-Luc containing binding sites for SMAD3 and SMAD4. mean ± SD of *n* = 6. *p* < 0.05 *, compared to PC-3 NTC; ^#^, compared to PC-3 shNQ; ^$^, compared to TGFβ-stimulated groups. **b** Immunoblotting of TGFβ response in nuclear extracts from shNTC and shNQ PC-3 cells. LaminB1 was used as a loading control for nuclear protein. All experiments were repeated a minimum of three times. **c** PC-3 NTC and PC-3 shNQ cells treated with TGFβ1 and LY2109761 for 48 h. Heatmap shows log2 gene expression changes detected by qPCR (*n* = 3). **d** Heatmap analysis from focused TGFβ qPCR array showing the differential gene expression between PC-3 NTC, PC-3 shNQ and PC-3 NTC + TGFβ1 treated groups (*n* = 2). See also Supplementary Figs. 9 and 10. **e** Validation of focused array by qPCR analysis with independent sets of specific primers. mean ± SD of *n* = 3 independent experiments. **f** Proposed schematic model showing how NQO1 suppresses TGFβ-induced EMT transition.

To test the hypothesis that NQO1 knockdown mimics activation of TGFβ signaling, we first determined TGFβ transcriptional activity using SBE4-Luc (four repeats of SMAD binding element; SBE). Promoter activity was significantly increased in shNQO1cells and was comparable to the effect of TGFβ1 treatment in NTC cells (*P* < 0.05). There was greater enhancement of luciferase activity in shNQO1 cells after TGFβ1 stimulation (Fig. 6a). LY2109761 (TGFBR1/2 inhibitor), inhibited TGFβ1-mediated SBE4-luc activation and attenuated NQO1 knockdown-mediated SBE4-luc activation in shNQO1 cells (Fig. 6a). Similarly, TGFβ transcriptional activity was also induced by NQO1 inhibition using MAC (Supplementary Fig. 7). We found the levels of SMAD3 and SMAD4 in the nucleus significantly increased in shNQO1 cells and these were further intensified in the presence of TGFβ1 (Fig. 6b and Supplementary Fig. 8; *P* < 0.05). TGFβ1 treatment reduced NQO1 gene expression in NTC cells while intensifying multiple TGFβ signature genes (Fig. 6c). The shNQO1 cells with higher TGFβ signature expression was inhibited by LY2109761 suggesting an interplay between NQO1 and TGFβ signaling (Fig. 6c). To characterize the extent of regulation of TGFβ signaling by NQO1, we used a focused qPCR-based array platform. Analyses of comparison between shNQO1 vs NTC and TGFβ1 vs NTC groups showed overlap of multiple TGFβ-associated genes (Fig. 6d). Additional analyses of the data using clustergram and scatter plot clearly showed that NQO1 knockdown mimics TGFβ signaling activation by regulating expression of multiple genes associated with TGFβ/BMP pathway (Supplementary Figs. 9 and 10). qRT-PCR validation with independent primers confirmed upregulation of TGFβ bonafide targets *SERPINE1*, *SMAD7* and *IGFBP3* and the downregulation of *FAS*. Knockdown of *NQO1* and suppression of *NQO1* by TGFβ1 treatment was also confirmed (Fig. 6e). As summarized in Fig. 6f, these results demonstrate that NQO1 suppresses TGFβ signaling pathway in prostate cancer cells and its suppression causes deleterious TGFβ activation perhaps by releasing the redox brake thus leading to advanced prostate cancer.

## Discussion

Prostate cancer is usually diagnosed as an androgen-dependent neoplasia. However, a subset of patients frequently progress to androgen-independence and metastasis to distant sites such as the bone, which constitutes a major cause of morbidity and mortality. Therefore, it is important to understand mechanisms involved, to identify targets for effective management of advanced prostate cancer. NQO1 appeard to play contextual roles in cancer tissues. For example, it is upregulated in various types of human cancers, and in many cases, is strongly associated with tumor progression and metastasis^19,35–37^. Colorectal cancer patients with elevated NQO1 expression have been shown to correlate with high-level of HIF-1α expression and poor prognosis^22^. Breast and cervical cancer patients with high-level NQO1 expression are also correlated with lower disease-free survival and 5-year overall survival rates compared with those with low-level NQO1 expression^21,38^. While these findings indicate that NQO1 is positively associated with tumor growth and malignant progression, NQO1 is also protective against carcinogens, essential for anti-ROS defense, quinone detoxification, and metabolic stress. Compounds that induce NQO1 expression are promising strategy for cancer chemoprevention^35,39^. NQO1 polymorphisms, particularly C609T that causes rapid ubiquitination and degradation of the protein^40^, have been implicated to have higher risk of several human cancers^41,42^.

Given the conflicting but critical role of NQO1 that influences cancer progression, we demonstrate that NQO1 knockdown cells rapidly develop into advanced prostate adenocarcinoma. However, it remains to be validated whether NQO1 knockdown potentiates metastatic disease burden in vivo. In the future, experimental models can be used to determine if NQO1 is causal for prostate cancer cells to home to different metastatic sites given our observations of heterogeneity in downstream signaling and functional output in prostate cancer cells isolated from different metastatic sites. Further, a cohort of 150 retrospective patient samples, about half of the carcinoma samples (versus a quarter in benign/PIN groups) showed either negative or low NQO1 score. Although we did not analyze whether negative staining was associated with NQO1 polymorphism, it has been suggested that such focal loss may be due to NQO1 polymorphism and possibly associated with disease progression as indicated by concurrent PTEN loss^20^. Comparison of recurrence-free survival of patients based on NQO1 staining scores did not reach significance possibly due to our small cohort size. Nevertheless, assessment of publicly available expression datasets consistently demonstrated significant correlation between decreased NQO1 transcripts and poorly differentiated metastatic tumors, which was in agreement with our experimental data^24,26–30^ (Fig. 2a; Supplementary Fig. 1). Remarkably, expression data showed significant association with disease-free survival. Biochemically recurrent prostate cancer (BCR), determined by increasing prostate specific antigen (PSA) level, often occurs following initial therapy for primary cancer. Among BCR patients (*n* = 23) in our cohort, we noted 65% patients had decreased NQO1 levels and two thirds of them (10 out of 15) had higher stage cancer (stage 3a and b). Further, we observed more than two third of CTCs had low NQO1 which was significantly correlated with BCR outcome. It will be of interest to independently verify this observation using larger patient cohorts. Together, our results indicate that low NQO1 is associated with advanced tumor stages and high levels of NQO1 may protect from tumor progression.

In many of the cohorts in public databases, decreased NQO1 transcript levels correlated with concurrent increase in expression of mesenchymal markers such as N-cadherin, fibronectin in metastasis^26,27,29,30^. Therefore, we assessed the role of NQO1 in prostate cancer metastasis using prostate cancer cell culture models (PC-3 NQO1 knockdown and ARCaP EMT model systems). These cellular models along with TGFβ-stimulated PC-3 model confirmed a role for NQO1 suppression in EMT progression. Our results are particularly related with metastasis since ARCaP_M_ is a lineage-derived from ARCaP_E_ cells that gained 100% incidence in bone and adrenal gland metastasis in mice^32^. PC-3 is a bone metastasis-derived cell line and TGFβ controls multiple signaling pathways associated with bone metastasis of prostate cancer cells^12^. Although we did not screen many markers of EMT changes, there was no consistent change in classic EMT markers in other prostate cancer cell lines with NQO1 knockdown (Supplementary Fig. 11). Additional work is needed to determine if NQO1 plays a role in the ability of cancer cells to home to different metastatic sites given our observations of heterogeneity in downstream signaling and functional output in prostate cancer cells. However, it is fascinating to note that NQO1 expression levels are significantly low in metastatic tissues derived from multiple sites of prostate cancer patients suggesting a possible gatekeeper role against metastasis (Fig. 2a).

Using a mouse model of nonalcoholic steatohepatitis (NASH), Sharma et al.^43^ showed that pharmacological activation of NRF2, a master regulator of NQO1, decreases TGFβ expression. However involvement of NQO1 itself or oxidative stress is not known. Our results show that decreased NQO1 in ARCaP_M_ clone increases TGFβ signaling and expression of mesenchymal genes, suggesting the operation of a negative feedback loop. Similarly, TGFβ signaling can be inhibited by NQO1 overexpression in the same clone. The data are consistent with increased TGFβ target genes as well as TGFβ-SMAD activation caused by NQO1 knockdown in PC-3 cells. These data provide direct evidence that NQO1 inhibits TGFβ signaling. We had reported that NQO1 silencing in hormone-responsive LNCaP cells enhanced several cytokines including IL-8 that reinforces cellular pro-migratory and pro-survival signaling^19^. These cytokines may potentially induce the transformation of prostate cancer cells including PC-3 cells to a more migratory/mesenchymal phenotype. Cytokine array analyses revealed that NQO1 knockdown in PC-3 cells decreased macrophage migration inhibitory factor (MIF) secretion (Supplementary Fig. 12). MIF is a pleotropic cytokine and oxidative stress sensor which modulates GSH levels by altering the cellular GSH/GSSG balance. It has recently been shown that MIF knockdown leads to the activation of the TGFβ-signaling^44,45^. The change in MIF further supports our notion that NQO1 silencing affects the redox balance and TGFβ signaling.

Over-treatment of low-risk prostate cancer contributed to recommendations against routine use of PSA screening. Improved risk assessment at the time of diagnosis may help reduce overtreatment by discriminating aggressive from indolent prostate cancer^46^. Deciphering NQO1 levels in cancer patients may be helpful to exploit the use of NQO1 targeted therapy^37^. On the other hand, low NQO1 levels not only show poor response to such therapies^46^ but also indicates disease progression (this study). Our results suggest that including NQO1 as a marker of disease progression (e.g., BCR) in molecular analyses and combining this with histopathological profiles could help stratify patients for aggressiveness of prostate cancer.

In summary, we show that NQO1 regulates TGFβ signaling to inhibit EMT and migration, which are required for prostate cancer progression. The proposed model (Fig. 6f), provides an explanation for how NQO1 overcomes the vulnerability to cellular stress, to obstruct prostate cancer cell plasticity that is essential for its progression to aggressive disease. We show that inhibition of NQO1 significantly correlated with EMT-like morphological changes and increased migration. This also increased TGFβ signaling and expression of multiple genes associated with prostate cancer progression and metastasis. NQO1 knockdown increased levels of nuclear SMADs thereby activating TGFβ-SMAD-ZEB1 signaling. Importantly, our biological data provide mechanistic details for the reported correlation between decreased NQO1 transcript and poorly differentiated metastatic prostate tumors and confirms our hypothesis that attenuation of NQO1 plays a role in TGFβ signaling mediated cancer cell plasticity.

## Methods

### Cell culture

Human androgen-repressed prostate cancer ARCaP_E_ (epithelial clone) and ARCaP_M_ (mesenchymal clone) were purchased from Novicure Biotechnology. Cells were used within eight passages of thawing. ARCaP cells were not authenticated but confirmed based on morphology, AR expression and EMT-associated genes. LNCaP and PC-3 cells were purchased from ATCC and not authenticated but were confirmed by expression or loss of AR, PSA and PTEN (Supplementary Fig. 13). ARCaP cells were cultured in MCaP-medium supplemented with 5% fetal bovine serum (FBS). LNCaP and PC-3 were cultured in RPMI1640 and F12-K, respectively. Cells were supplemented with 10% FBS, 1% antibiotics and antimycotics (GibcoBRL), and maintained in 5% CO_2_ at 37 °C.

### Stable cell line generation

Stable NQO1 knockdown was generated as previously published^19^. Briefly, lentiviral particles expressing shRNA against non-targeted control (NTC) and NQO1 (shNQ) were purchased from Dharmacon (Lafayette, CO). LNCaP and PC3 cells were transduced with either NTC or shNQ in the presence of 8 μg/ml polybrene (Sigma-Aldrich) in 96-well plate. Transduction was continued for 48 h. Cells were transferred into a 24-well plate and selected with 1 μg/ml puromycin (Sigma-Aldrich). Serially diluted cells were grown and efficiency of transduction was confirmed in isolated clones by detecting GFP fluorescence. For transient knockdown, cells were transfected with either scramble or NQO1 SMARTpool siRNA (Dharmacon) using Lipofectamine (Invitrogen). Knockdown was verified by qPCR and immunoblot analysis. For stable overexpression, cells were transfected with pcDNA3.1 vector control or pcDNA-NQO1 (pcNQ). After selection with G418, NQO1 overexpression was verified by western blotting.

### Animal experiments

For orthotopic implantation of human prostate cancer cells, male athymic nude mice (4–6 weeks of age) were obtained from Charles River Laboratories. After one-week acclimation period, mice were randomized into two groups (*n* = 8 per group) and implanted orthotopically with 1 × 10^6^ LNCaP cells either expressing stably silenced NQO1 (LNCaP shNQ) or non-targeted control (LNCaP NTC) in matrigel (1:1 v/v, Corning). Tumor development was examined by palpation at the site of injection weekly and body weight was measured weekly. The experiment was terminated when the first palpable tumors were noted irrespective of group. Mice were euthanized and all tissues including serum were collected. One animal in the shNQ group died from non-tumor related cause. The prostates were observed for gross tumor, weighed and photographed. A portion of each prostate tissue was snap-frozen and the remaining formalin-fixed and paraffin-embedded. Following Hematoxylin and Eosin staining of the embedded tissue, histopathological evaluation was conducted by a blinded pathologist. All animal procedures were approved by UTHSCSA IACUC.

### Bioinformatics data analysis

Microarray dataset of NQO1 knockdown and nontargeted control LNCaP cells (GSE58336) were analyzed by DAVID web tool. The Cancer Genome Atlas (TCGA) and other publicly available datasets were accessed. Expression of NQO1 in primary and metastatic patient samples was queried using Oncomine database (Chandran^24^, Yu^26^, Grasso^25^, Lapointe^27^, Ramaswamy^28^, Ramaswamy2^29^, and Tamura^30^ prostate studies available through Oncomine http://www.oncomine.org/). Kaplan–Meier analysis was extracted (GSE40272; GSE70769) and analyzed from PROGgeneV2. Cluster heatmap data were accessed and analyzed using cBioPortal for TCGA and SU2C/PCF Dream Team.

### Prostate tumor specimens and immunohistochemical analysis

A set of TMA consisting of 150 radical prostatectomy specimens was constructed as described previously at the University of Rochester Medical Center^47^. Appropriate approval was obtained from the URMC institutional review board for the construction and use of the TMA. The tissue sections were immunohistochemically stained with anti-NQO1 along with relevant controls as described previously^19^. Immunoreactive scores were calculated as negative (0) weakly positive (1+), moderately positive (2+), and strongly positive (3+), as described earlier^47^, via considering both staining intensity and the percentage of Immunoreactive cells. Recurrence-free survival was compared between high (2/3+) and low NQO1 (0/1+) expression groups.

### Circulating tumor cells (CTCs)

Subjects were enrolled in a longitudinal, observational clinical study called Longitudinal Study of Prostate Cancer Determinants of Resistance (Los Padres). After obtaining UTHSCSA IRB consent, we obtained blood (8-16 mL per patient) for CTC isolation with the following modification^48^. The peripheral blood mononuclear cell (PBMC) interphase portion was collected following Ficoll cushion centrifugation. MojoSort™ Human CD45 Nanobeads (BioLegend, #480030) was used to deplete CD45^+^ cells. To enrich CTCs, we cultured cells in PRIME-XV tumorsphere medium (Irvine Scientific, #91130) and then expanded into tumor spheres for 1 to 2 weeks. A nanobead depletion assay was carried out to remove CD45-positive cells before cDNA generation.

### Single-cell transcriptomic sequencing, mapping, and clustering of CTCs

Viable cells (detected with LIVE/DEAD Viability/Cytotoxicity Kit; Invitrogen) were loaded in Fluidigm C1 single-cell mRNA seq IFC (Fluidgm) for single-cell isolation. Single-cell cDNA synthesis was carried out with SMART-Seq v4 Ultra Low Input RNA Kit (Clontech). Single-cell cDNA synthesized from each living cell was used in RNA-seq library preparation per the manufacturer’s protocol. Nextera XT DNA Library Prep Kit (Illumina) with Nextera XT Index Kit v2 Set A and B index primers were used for cDNA tagmentation using diluted cDNA (0.3 ng/μL). Paired-end cDNA sequencing was carried out on the HiSeq 3000 system (Illumina). Base calling was performed using the Illumina Real-Time Analysis software. Output paired-end FASTQ files were aligned to the GRCh38 reference genome using STAR alignment software^49^. Gene expression levels were quantified by applying RSEM^50^, and fragments per kilobase of transcript per million (FPKM) mapped reads were calculated. Cells with four leucocyte markers (displaying low uniquely aligned reads and high expression; PTPRC, CD34, CD19, and FCGR3A) were excluded from further analysis. t-Distributed Stochastic Neighbor Embedding (t-SNE) (R package Rtsne) was used to generate scatter plot of 136 CTCs based on NQO1 and TGFβ transcriptomic profile (log FPKM + 1), and mclust algorithm was subsequently applied to robustly differentiate CTCs into different clusters.

### Cell viability, colony formation, and migration assays

Cell viability and colony formation assays were performed as described previously^19^. Briefly, the MTT assay is used to determine the cell viability. Absorbance was measured and normalized to express cell viability in percent of control. To assess colony formation 1 × 10^3^–5 × 10^3^ cells were seeded in triplicate in 6-well plate. After 9–12 days, colonies were fixed, stained with 0.05% crystal violet in methanol. Plates were scanned to obtain digital images. To determine chemotactic migration through a 8 µm pore size insert (Corning), 50 µL cell suspension (5 × 10^4^ cells in serum free medium) was dispensed in the upper insert and allowed to migrate towards 10% serum containing medium on the bottom well.

### Cytokine array

Antibody-based array was used to determine cytokines in culture supernatant according to the manufacturer's protocol (R&D Systems).

### RNA extraction and real-time quantitative PCR (qPCR)

Total RNA was extracted and used to transcribe into cDNA as published^21^. Cells were harvested and total RNA was extracted using TRIzol according to the manufacturer’s instructions (Invitrogen, Carlsbad, CA). Isolated RNA was reverse transcribed into cDNA using SuperScript VILO cDNA Synthesis Kit (Invitrogen). Primer pairs (Supplementary Table 4) were purchased from Integrated DNA Technologies. Gene expression levels were analyzed by qPCR on CFX96 Real-Time PCR Detection System (Bio-Rad Laboratories, CA). A standard program: 95 °C for 10 min, 40 cycles at 95 °C for 15 s and 60 °C for 1 min was used for all qPCR assays. An additional melting step was added to analyze the specificity of the obtained amplification products. Each Ct value was normalized to β-actin levels as reference.

### Transient transfection and luciferase activity

Stable clones of PC-3 NTC and PC-3 shNQ were co-transfected with SBE4-Luc^51^ (Addgene) and pRL-TK (Promega) using Lipofectamine 2000 according to the manufacturer’s instruction (Invitrogen). Cells were plated at a density of 1 × 10^5^ cells/well in 24-well plate. After 24 h, cells were transfected with 0.5 µg of promoter-linked luciferase vector and 10 ng of pRL-TK vector in OptiMEM medium (Invitrogen). Luciferase activity was determined using the dual luciferase reporter assay (Promega, Madison, WI), and emitted light was measured with a luminometer (Turner Biosystems, Sunnyvale, CA).

### qPCR array

TGFβ gene expression was analyzed using qPCR arrays (Human TGFβ/BMP Signaling Pathway RT^2^ Profiler™ PCR Array, QIAGEN, Cat. # PAHS-035Y) on CFX96 Real-Time PCR Detection System per manufacturer’s instructions.

### Western blot analysis

Whole cell extracts were prepared using 2 × SDS-containing-Laemmli buffer. Cytoplasmic and nuclear extracts were prepared using the NE-PER nuclear extraction kit (Pierce). Equal amounts of total, cytoplasmic or nuclear extracts were resolved by SDS-PAGE and immunoblotted with specific primary antibodies. Primary antibodies were diluted in 5% non-fat milk or 3% bovine serum albumin (BSA) in Tris-buffered saline with 0.1% Tween-20 (TBS-T) at the following dilutions: 1:500 (PTEN, HDAC1, Fibronectin); 1:1000 (AR, α-tubulin, TCF8/ZEB1, N-cadherin); 1:2000 (PSA, E-cadherin, Vimentin, β-catenin, Smad2, Smad3, Smad2/3, Smad4); 1:7500 (NQO1, β-actin, LaminB1). Proteins were visualized using ECL kit (Perkin Elmer) and digitally processed using Syngene G-Box (Syngene, Frederick, MD). Membranes were stripped and re-probed with β-actin and Lamin B1 antibodies for total and nuclear loading controls respectively.

### Immunofluorescence

Cells were grown on cell culture-treated coverglass and immunostained with specific antibodies at the following dilution: 1:100 (N-cadherin); 1:250 (E-cadherin, Vimentin) and 1:500 (NQO1). Immunofluorescence was performed as described previously^19^. Immunostained cells were examined on a Sweptfield confocal system (Prairie Technologies, Middleton, WI) equipped with a Nikon Ti microscope.

### Statistics and reproducibility

Measurements were made on distinct samples. Difference between groups was analyzed by ANOVA or Student’s *t*-test using GraphPad Prism software. TGFβ qPCR array was performed twice. For all other in vitro assays, three independent experiments were performed to confirm reproducibility. In clinical samples and public data sets, sample size was limited by availability. In in vivo mouse and in vitro assays, sample sizes were chosen based on our experience and previously published literature. Fisher's exact test was used to analyze significance of the associations between disease grade, clinicopathological features and NQO1 staining levels. For survival analysis, Kaplan–Meier curves were generated using either PROGgeneV2 or Prism software and log-rank analysis was performed. Results are expressed as mean ± SD. *P* values < 0.05 were considered statistically significant.

### Reporting summary

Further information on research design is available in the Nature Research Reporting Summary linked to this article.

## Supplementary information

Supplemental Information

Supplementary Data 1

Description of Additional Supplementary Files

Reporting Summary

## Data Availability

Our microarray data set of NQO1 knockdown and non-targeted control LNCaP cells (GSE58336) and RNA sequencing data from CTCs (GSE115501) are available in Gene Expression Omnibus (GEO) (https://www.ncbi.nlm.nih.gov/geo/). NQO1 expression in patient samples from multiple cohorts referenced in the study are available in Oncomine (http://www.oncomine.org/). The TCGA and SU2C/PCF data are publicly available (http://www.cbioportal.org/). Kaplan–Meier analysis of two cohorts (GSE40272; GSE70769) are available in PROGgeneV2 (http://www.compbio.iupui.edu/proggene). All other data supporting the findings of this study are available within the article and its supplementary information files. Raw data are provided as Supplementary Data 1.
