# Supplementary material for: Attenuation of NAD[P]H:quinone oxidoreductase 1 aggravates prostate cancer and tumor cell plasticity through enhanced TGFβ signaling
Source: Commun Biol. 2020 Jan 3;3:12. doi: 10.1038/s42003-019-0720-z (PMC6941961; doi:10.1038/s42003-019-0720-z)
Supplement: Supplementary file 1 — Supplemental Information [file 42003_2019_720_MOESM1_ESM.pdf]

# Supplementary Figures

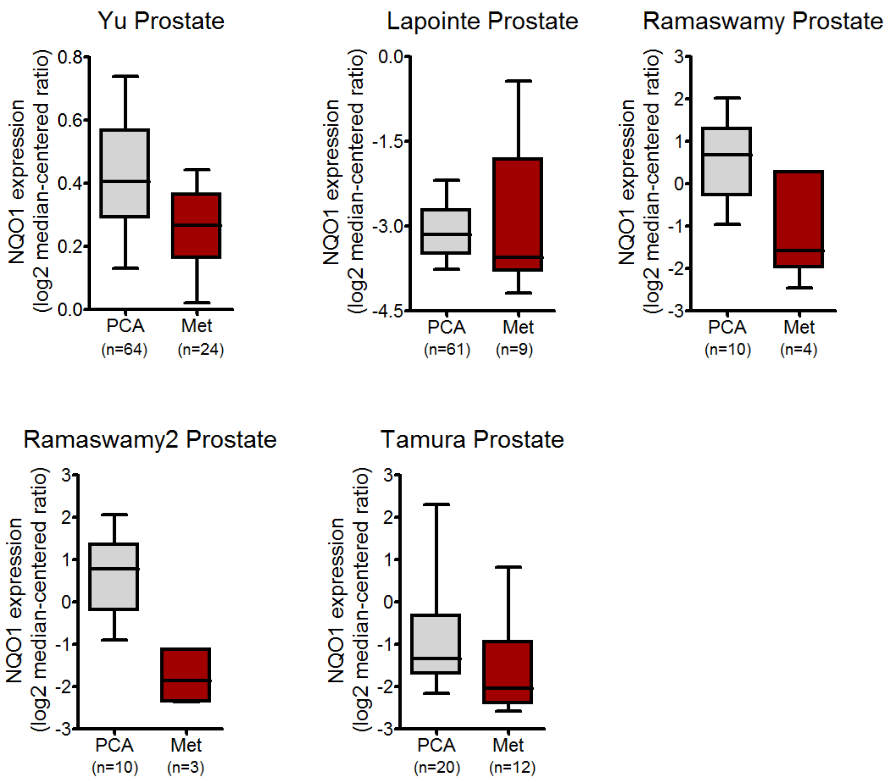


**Supplementary Fig. 1:** NQO1 expression in human prostate cancer. The changes in NQO1 expression levels between human prostate cancer and metastasis. The data sets are based on Yu, Lapointe, Ramaswamy, Ramaswamy2 and Tamura prostate studies from Oncomine.

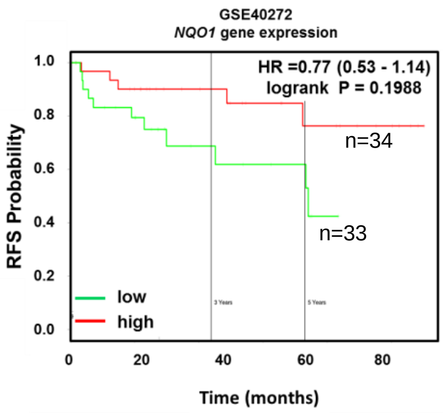


**Supplementary Fig. 2:** Kaplan-Meier analysis of recurrence-free survival (RFS) was compared between high and low NQO1 transcript levels using median gene expression value as a bifurcating point. RFS plot of 67 patients from cohort GSE40272.

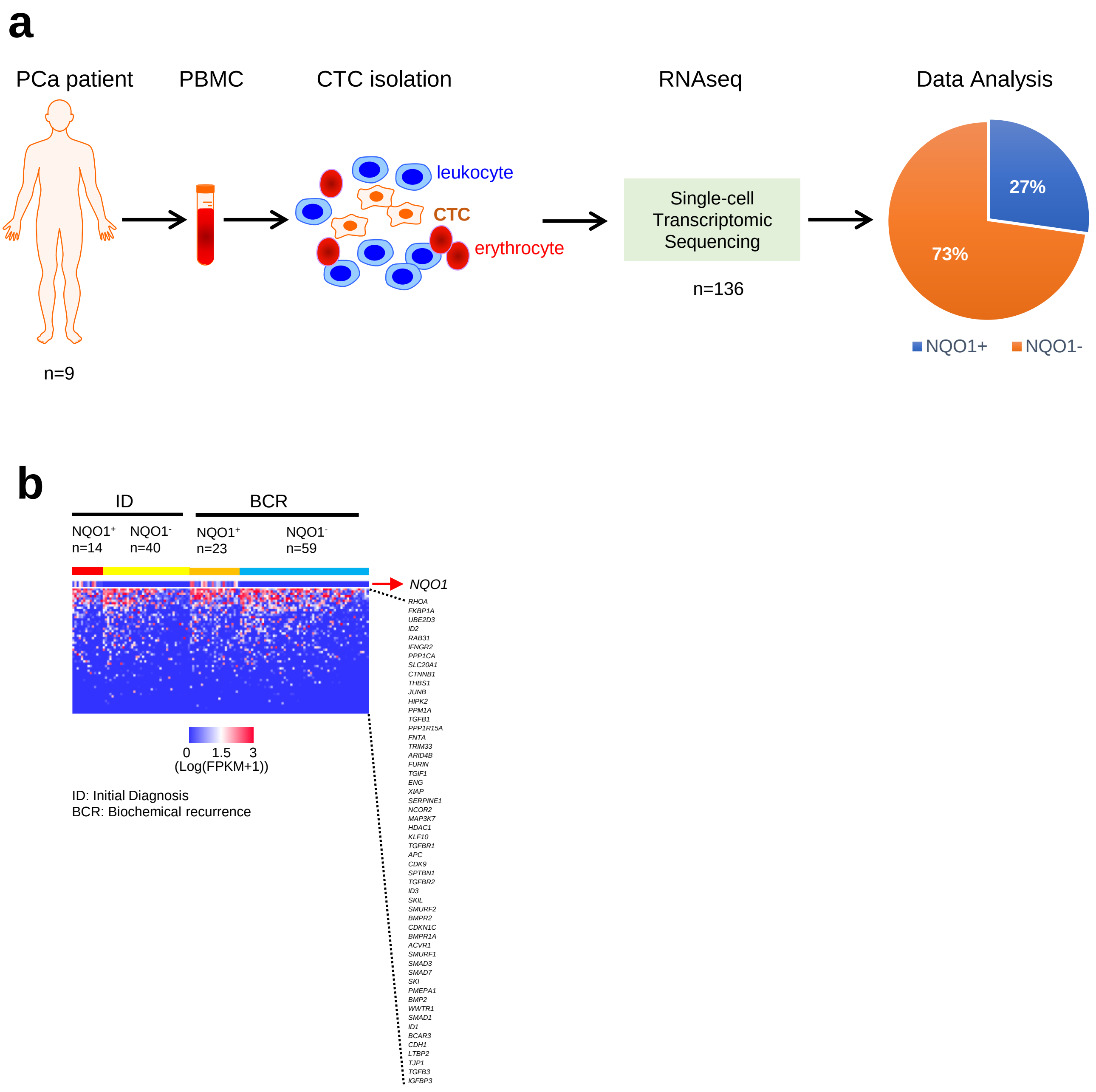


**Supplementary Fig. 3:** (a) Circulating Tumor Cells (CTCs) were isolated from patients with prostate cancer (n=9) and single-cell RNA sequencing expression analyzed for NQO1. The schematic images of human body, test tube and cells were prepared using the Biomedical-PPT-Toolkit-Suite (Motifolio Inc., USA). (b) Heat maps of *NQO*1 and TGF-β pathway expression profile.

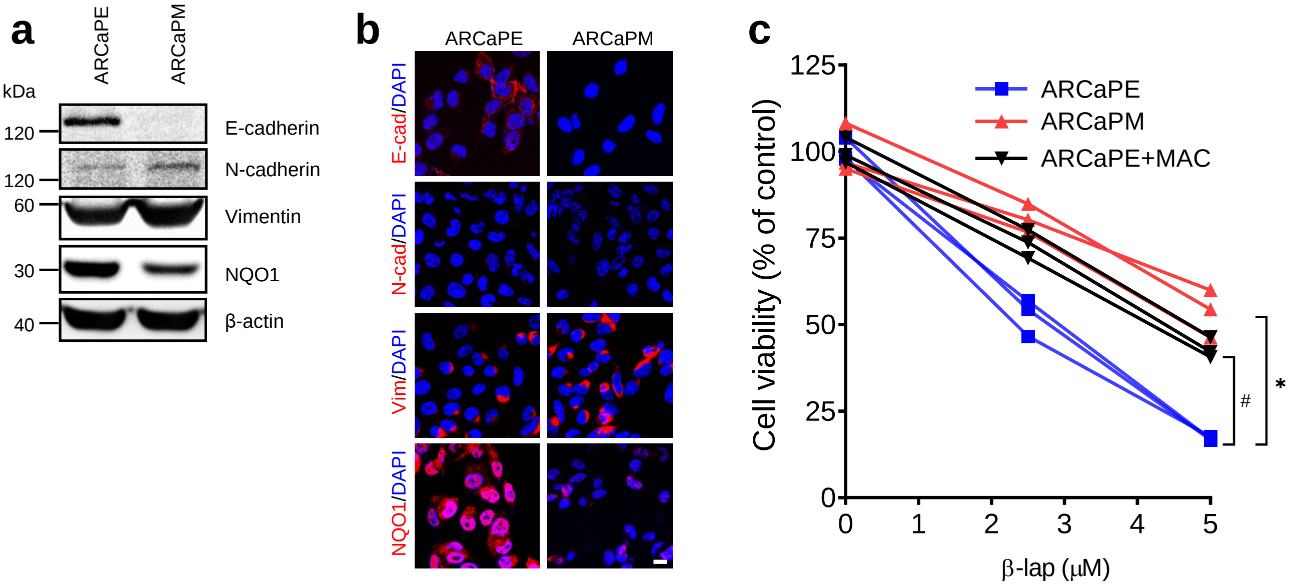


**Supplementary Fig. 4:** (**a**) Western blot analysis in whole cell lysates. (**b**) Representative immunofluorescence images of fixed cells. Representative images shown are from two independent experiments performed in duplicate wells. Scale bar = 20 μm (**c**) MTT assay was performed to assess cell viability in the presence of β-lap, MAC220 and the combination. Differential response to β-lap between $ARCaP_E$ and $ARCaP_M$ at the tested concentration (*, *P*<0.05). MAC220 partially reversed the effect of β-lap in $ARCaP_E$ cells (#, *P*<0.05 with unpaired t-test). The experiment was repeated thrice in duplicate.

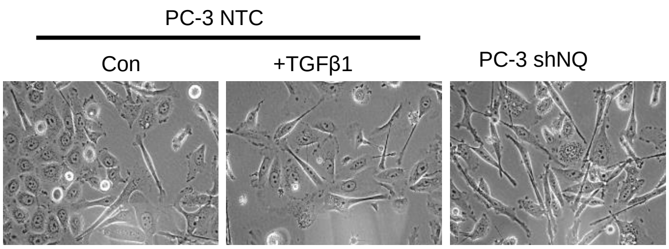


**Supplementary Fig. 5:** TGFβ1-treated PC-3 NTC cells mimic mesenchymal-like phenotype of PC-3 shNQ cells.  PC-3 NTC cells were treated with TGFβ1 (2 ng/ml for 72 h) and images were taken (20x). Representative images show the change in morphology of PC-3 cells with or without TGFβ1 stimulation for undergoing EMT.

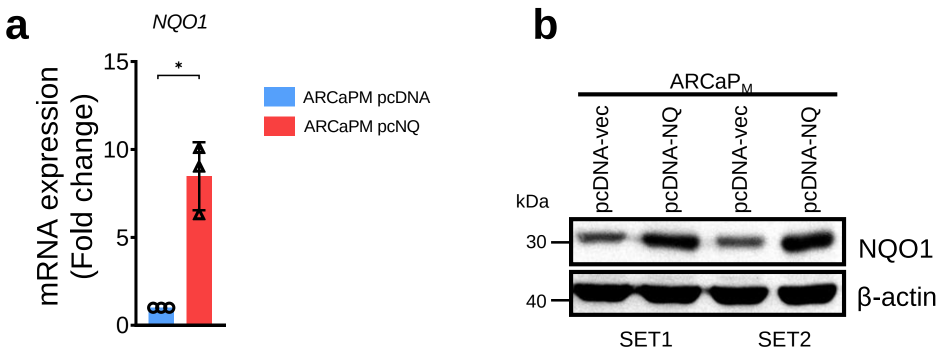


**Supplementary Fig. 6:** Stable NQO1 overexpression in $ARCaP_M$ cells. pCDNA3.1 vector or pcDNA3-NQO1 construct was transfected into the $ARCaP_M$ cells. The stable clones were selected using G418. RT-qPCR (**a**) and western blot (**b**) analyses were performed to confirm the overexpression. (mean±SD of n=3 independent experiments. *, $P<0.05$; *t*-test).

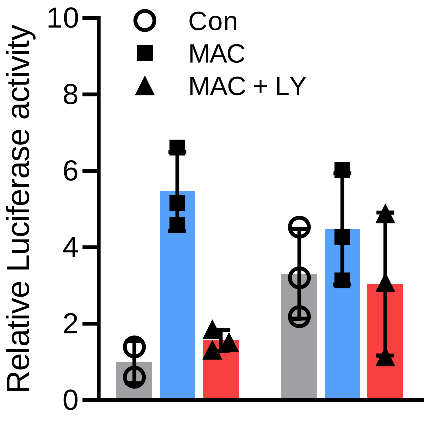


**Supplementary Fig. 7:** PC-3 NTC and PC-3 shNQ cells were transiently transfected with SBE4-Luc containing binding sites for SMAD3 and SMAD4. NQO1 inhibitor MAC (500 nM) treatment stimulated reporter luciferase activity and LY2109761 (5 µM) inhibited MAC-mediated induction of luciferase activity.

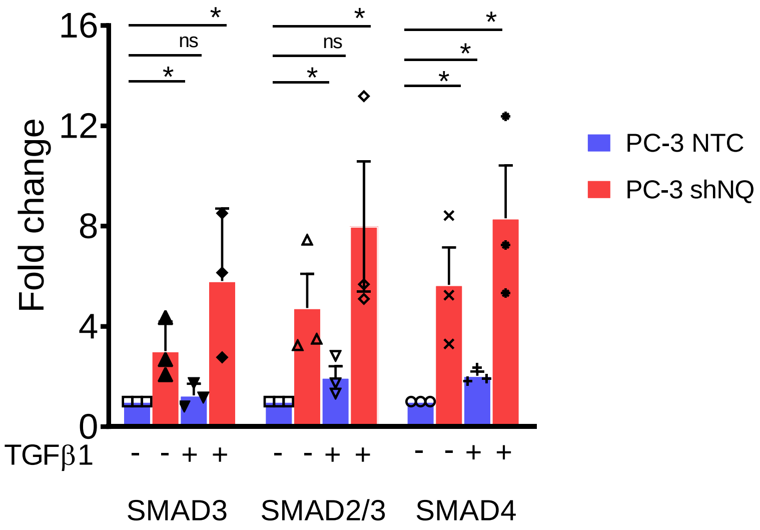


**Supplementary Fig. 8:** Quantification of nuclear SMADs. PC-3 NTC and PC-3 shNQ cells were treated with TGFβ1 for 1 h. The cytoplasmic and nuclear proteins were extracted using NE-PER kit and subjected to western blot analysis for nuclear levels of SMAD3, SMAD2/3 and SMAD4 using specific antibodies. HDAC1 and LaminB1 were used as normalizing controls. mean±SD of n=3 independent experiments. (*, $P<0.05$; *t*-test).

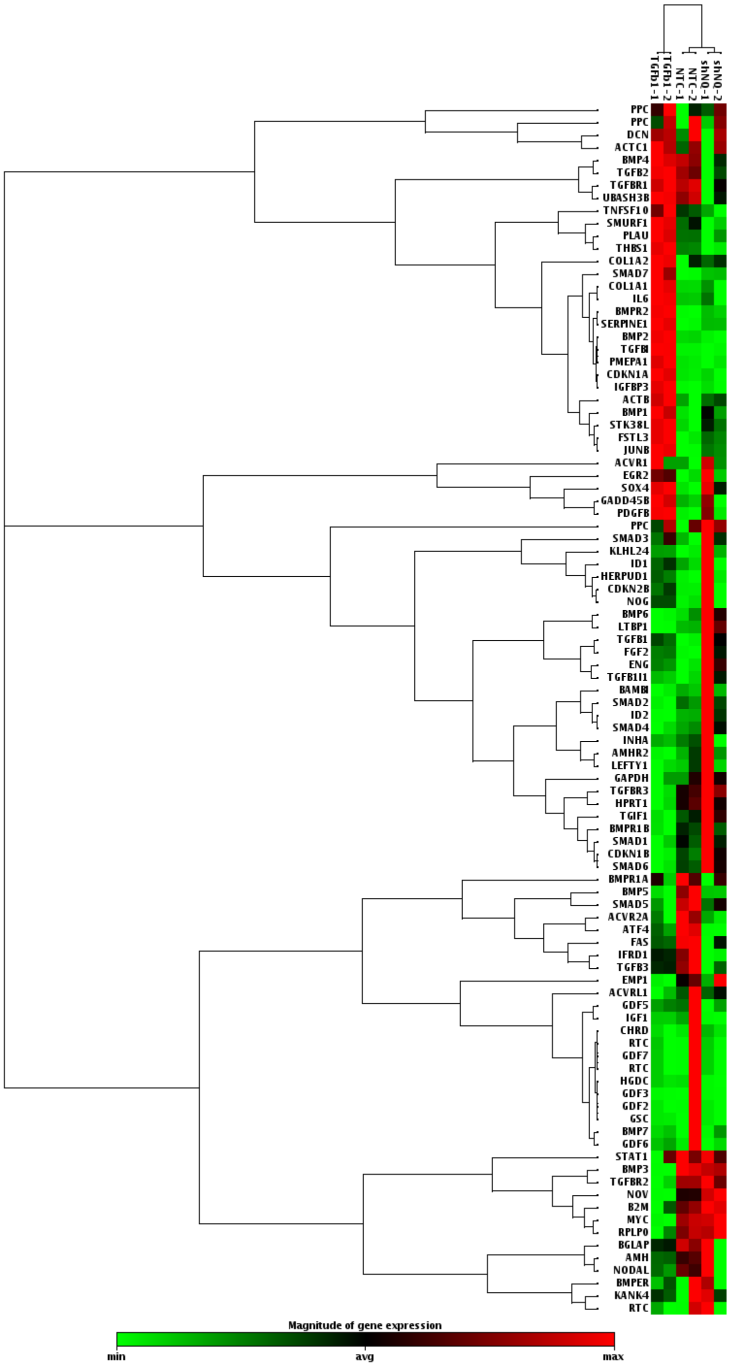


**Supplementary Fig. 9:** Non-supervised hierarchical clustering of the qPCR array. Heat map with dendrograms indicating co-regulated genes across three groups with two individual samples.

**a**

**Arrays included in Test Group:** **shNQ-1, shNQ-2**
**Arrays included in Control Group:** **NTC-1, NTC-2**

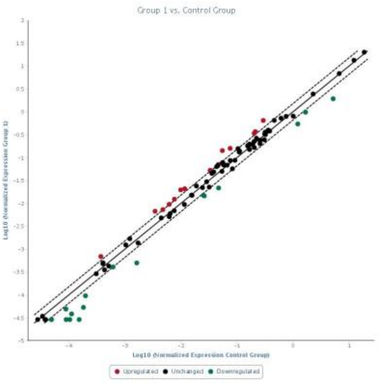


**Genes Over-Expressed in shNQ vs NTC**

| Gene Symbol | Fold Regulation |
|---|---|
| FGF2 | 2.58 |
| SERPINE1 | 2.19 |
| ID2 | 2.10 |
| ENG | 1.99 |
| SMAD7 | 1.95 |
| BMP6 | 1.86 |
| TGFB1I1 | 1.79 |
| JUNB | 1.73 |
| TGFB1 | 1.67 |
| PDGFB | 1.61 |
| BAMBI | 1.58 |
| SMAD6 | 1.55 |
| SOX4 | 1.54 |
| EGR2 | 1.54 |
| KLHL24 | 1.52 |

**Genes Under-Expressed in shNQ vs NTC**

| Gene Symbol | Fold Regulation |
|---|---|
| IGF1 | -5.25 |
| HGDC | -3.57 |
| BMP5 | -3.25 |
| THBS1 | -2.71 |
| TGFB2 | -2.24 |
| FAS | -2.17 |
| CHRD | -2.06 |
| BMP7 | -1.80 |
| BMP4 | -1.79 |
| PLAU | -1.71 |
| TGFBI | -1.68 |
| BMP2 | -1.50 |

**b**

**Arrays included in Test Group:** **TGFb1-1, TGFb1-2**
**Arrays included in Control Group:** **NTC-1, NTC-2**

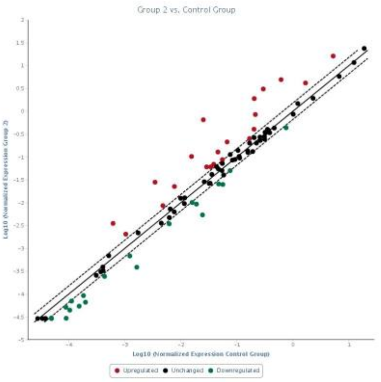


**Genes Over-Expressed in TGFβ1 vs NTC control**

| Gene Symbol | Fold Regulation |
|---|---|
| TGFBI | 25.86 |
| SERPINE1 | 10.36 |
| PMEPA1 | 9.24 |
| SMAD7 | 8.13 |
| IGFBP3 | 7.99 |
| CDKN1A | 6.60 |
| BMP2 | 5.73 |
| JUNB | 3.95 |
| IL-6 | 3.18 |
| THBS1 | 3.07 |
| PDGFB | 2.94 |
| FSTL3 | 2.79 |
| PLAU | 2.47 |
| COL1A1 | 2.10 |
| EGR2 | 1.80 |
| SOX4 | 1.77 |
| FGF2 | 1.58 |

**Genes Under-Expressed in TGFβ1 vs NTC control**

| Gene Symbol | Fold Regulation |
|---|---|
| BMP3 | -4.51 |
| BMP5 | -4.21 |
| CHRD | -2.97 |
| IGF1 | -2.80 |
| HGDC | -2.33 |
| FAS | -1.84 |
| SMAD6 | -1.79 |
| LEFTY1 | -1.79 |
| MYC | -1.74 |
| BMP7 | -1.71 |
| ID2 | -1.51 |

**Supplementary Fig. 10:** Scatter plot showing over-expressed and under-expressed genes. Normalized gene expression of PC-3 NTC vs PC-3 shNQ (**a**) and NTC vs TGFb1 group (**b**). Scatter plot shows genes overexpressed and underexpressed. The central line indicates unchanged gene expression. The dotted lines indicate the selected fold regulation threshold 1.5 fold. Selected genes that are overexpressed and underexpressed are listed in Tables on right.

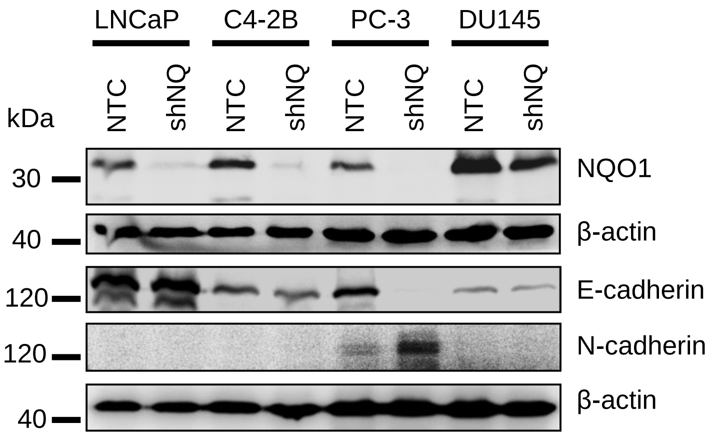


**Supplementary Fig. 11:** Stable NQO1 knockdown and classical EMT markers (E-cadherin and N-cadherin) in prostate cancer cell lines. All cells were cultured in respective complete media for 2-3 days. Whole cell lysates were prepared and subjected to western blot analysis using indicated antibodies to confirm the expression of specific markers. β-actin is used as a loading control (n=2).

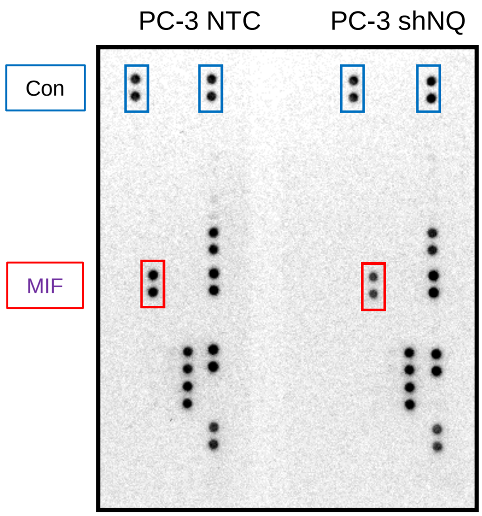


**Supplementary Fig. 12:** Cytokine array: PC-3 NTC and PC-3 shNQ cells were cultured in standard medium. Conditioned media derived from these cells for 24 h were subjected to cytokine expression profiling using human cytokine expression array.

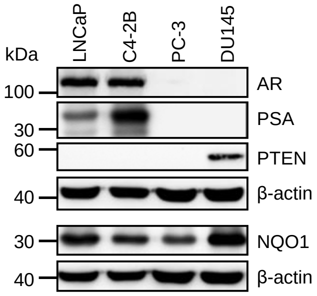


**Supplementary Fig. 13:** AR, PSA, PTEN and NQO1 protein levels in prostate cancer cell lines. All cells were cultured in respective complete media for 2-3 days. Whole cell lysates were prepared and subjected to western blot analysis using indicated antibodies to confirm the expression of specific markers. The blots are representative of three technical repeats of different passages. β-actin is used as a loading control.

## Supplementary Tables

**Supplementary Table 1**: Grading Scheme

| Grade | Histology | Representative H&E sections |
|---|---|---|
| Grade 0 | • Dilated acini<br>• Flattened epithelial border<br>• Papillary formation that does not fill the acinus<br>• No cellular pleomorphism<br>• No mitotic activity | 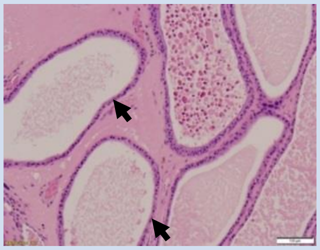 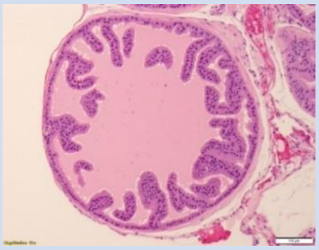 |
| Grade 1 | • Short to elongated papillary projections that partially fill the lumen of the acinus<br>• Flat epithelial lining in areas between papillary structures<br>• Slight increase in bland nuclei within the papillary stalk, rare<br>• Nuclei may be stratified and have a bland morphology<br>• No cellular pleomorphism<br>• No mitotic activity<br>• May coexist with other grades | 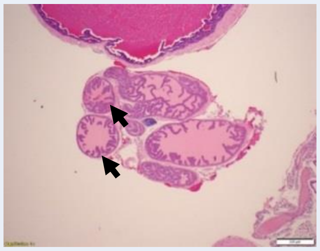 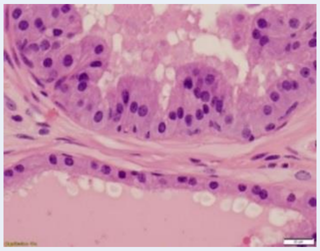 |
| Grade 2 | • Variable size acini, small to large<br>• Papillary projections that do not completely fill the lumen of the acinus<br>• Rare papillary structures with increased numbers of nuclei<br>• No nuclear pleomorphism<br>• No mitotic activity<br>• May coexist with other grades | 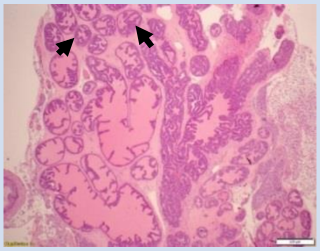 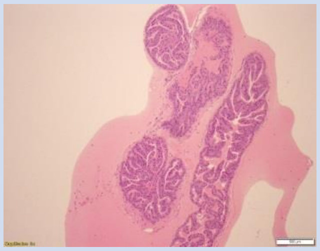 |
| Grade 3 | • Variable size acini, mostly large and many are elongated<br>• Papillary projections fill the lumen of the acinus<br>• Increased numbers of nuclei within the acinus<br>• May co-exist with lower morphological grades<br>• Nuclei are bland in most areas with minimal nuclear pleomorphism<br>• Mitoses are absent | 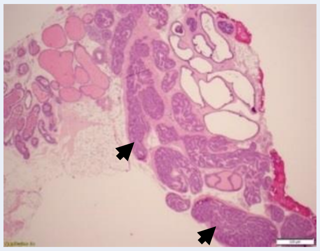 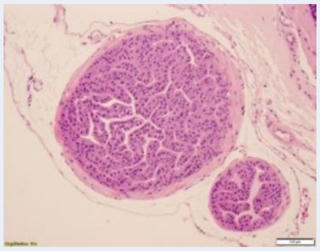 |

## **Supplementary Table 2**: Analysis of percentage of NQO1 staining

| | N | NQO1 low 0/1+ (%) | NQO1 high 2+/3+ (%) | Fisher test (*P* value) 0/1+ *vs.* 2+/3+ |
|---|---|---|---|---|
| Benign | 125 | 38 (30.4) | 87 (69.6) | 0.347 |
| PIN | 123 | 45 (36.6) | 78 (63.4) | 0.038[*2] |
| Carcinoma | 150 | 74 (49.3) | 76 (50.7) | 0.002[*1] |

[*1]Benign *vs.* Carcinoma

[*2]PIN *vs.* Carcinoma

## **Supplementary Table 3**: Analysis of clinicopathological data

| Clinicopathologic features | N | 0 (%) | 1+ (%) | 2+ (%) | 3+ (%) | Fisher test (*P* value) | | | Score | Student's *t*-test |
|---|---|---|---|---|---|---|---|---|---|---|
| | | | | | | 0 *vs.* 1+/2+/3+ | 0/1+ *vs.* 2+/3+ | 0/1+/2+ *vs.* 3+ | (mean ± SD) | (*P* value) |
| **Gleason score (GS)** | | | | | | 0.465[*1] / 0.472[*2] | 1.000[*1] / 0.622[*2] | 0.533[*1] / 1.000[*2] | | 0.892[*1] / 0.679[*2] |
| **≤6** | 60 | 6 (10.0) | 24 (40.0) | 16 (26.7) | 14 (23.3) | | | | 5.34 ± 3.34 | |
| **7** | 72 | 12 (16.7) | 22 (30.6) | 26 (36.1) | 12 (16.7) | | | | 5.33 ± 3.28 | |
| **≥8** | 18 | 1 (5.6) | 9 (50.0) | 4 (22.2) | 4 (22.2) | | | | 5.00 ± 3.19 | |
| **Pathologic stage (pT)** | | | | | | 0.269[*3] / 0.569[*4] | 0.578[*3] / 0.572[*4] | 1.000[*3] / 0.814[*4] | | 0.747[*3] / 0.609[*4] |
| **2** | 111 | 12 (10.8) | 41 (36.9) | 36 (32.4) | 22 (19.8) | | | | 5.38 ± 3.18 | |
| **2+** | 2 | 1 (50.0) | 0 (0) | 1 (50.0) | 0 (0) | | | | 3.50 ± 3.54 | |
| **3a** | 28 | 4 (14.3) | 10 (35.7) | 7 (25.0) | 7 (25.0) | | | | 5.39 ± 3.74 | |
| **3b & 4** | 9 | 2 (22.2) | 4 (44.4) | 2 (22.2) | 1 (11.1) | | | | 4.33 ± 3.09 | |
| **Lymph node metastasis (pN)** | | | | | | 1.000[*5] | 1.000[*5] | 0.127[*5] | | 0.403[*5] |
| **0** | 92 | 14 (15.2) | 32 (34.8) | 30 (32.6) | 16 (17.4) | | | | 5.09 ± 3.19 | |
| **1** | 7 | 1 (14.3) | 2 (28.6) | 1 (14.3) | 3 (42.9) | | | | 6.43 ± 3.85 | |
| **x** | 51 | 4 (7.8) | 21 (41.2) | 15 (29.4) | 11 (21.6) | | | | 5.50 ± 3.38 | |

[*1]GS ≤6 *vs.* GS ≥7

[*2]GS ≤7 *vs.* GS ≥8

[*3]pT2 & pT2+ *vs.* pT3-4

[*4]pT2 *vs.* pT2+ & pT3-4

[*5]pN0 *vs.* pN1

**Supplementary Table 4**: Primer sequence

| Primer | F/R | Sequence |
|---|---|---|
| NQO1 | F | AAGGATGGAAGAAACGCCTGGAGA |
| | R | GGCCCACAGAAAGGCCAAATTTCT |
| Actin | F | GGCACCCAGCACAATGAAGATCAA |
| | R | TAGAAGCATTTGCGGTGGACGATG |
| E-cadherin | F | ACACTGCCAACTGGCTGGAGATTA |
| | R | TGATTAGGGCTGTGTACGTGCTGT |
| Vimentin | F | AGATGGCCCTTGACATTGAG |
| | R | TGGAAGAGGCAGAGAAATCC |
| ZEB1 | F | TACCTGTGAATGGGCGACCAAGAA |
| | R | ACTGCCTGGTGATGCTGAAAGAGA |
| TGFB1 | F | CATCCTAGACCCTTTCTCCT |
| | R | CTCAGTATCCCACGGAAATAAC |
| TGFB3 | F | TGGACTTCGGCCACATCAAGAAGA |
| | R | TGTTGTAAAGGGCCAGGACCTGAT |
| TGFBR1 | F | TCACTCATGTTGATGGTCTATATC |
| | R | CAACGTAGTACCCTCTGAAATAA |
| TGFBR2 | F | TATTAGAGAGGGACTGGTAGTG |
| | R | TTTCCAAACCTTTGCATGTATC |
| LTBP | F | CAACACTGTGGGCTCTTATC |
| | R | CACACTCCCAGATTGTCATC |
| IGFBP3 | F | CCAAACCCAAGAAGGTCTGGCAAA |
| | R | TTCAAGGAGAGCTCTATGCAGCGT |
| FAS | F | GGAAGGAGTACACAGACAAAG |
| | R | TCCGGGTGCAGTTTATTTC |
| SERPINE1 | F | GGGTGAAGACACACACAAA |
| | R | CGTTGAAGTAGAGGGCATTC |
| SMAD7 | F | GGACACCCTGATAGGAAGA |
| | R | CACACTCCTGACAAGTGAAA |
