# Supplementary material for: Attenuation of NAD[P]H:quinone oxidoreductase 1 aggravates prostate cancer and tumor cell plasticity through enhanced TGFβ signaling
Source: Commun Biol. 2020 Jan 3;3:12. doi: 10.1038/s42003-019-0720-z (PMC6941961; doi:10.1038/s42003-019-0720-z)
Supplement: Supplementary file 3 — Description of Additional Supplementary Files [file 42003_2019_720_MOESM3_ESM.pdf]

Description of Additional Supplementary Files

File Name: **Supplementary Data 1**
Description: Raw data in support of experimental results
