# Supplementary material for: Attenuation of NAD[P]H:quinone oxidoreductase 1 aggravates prostate cancer and tumor cell plasticity through enhanced TGFβ signaling
Source: Commun Biol. 2020 Jan 3;3:12. doi: 10.1038/s42003-019-0720-z (PMC6941961; doi:10.1038/s42003-019-0720-z)
Supplement: Supplementary file 4 — Reporting Summary [file 42003_2019_720_MOESM4_ESM.pdf]

nature research

Corresponding author(s): Rita Ghosh

Last updated by author(s): November 18, 2019

Reporting Summary

Nature Research wishes to improve the reproducibility of the work that we publish. This form provides structure for consistency and transparency in reporting. For further information on Nature Research policies, see Authors & Referees and the Editorial Policy Checklist.

Statistics

For all statistical analyses, confirm that the following items are present in the figure legend, table legend, main text, or Methods section.

n/a	Confirmed	
☐	☒	The exact sample size (n) for each experimental group/condition, given as a discrete number and unit of measurement
☐	☒	A statement on whether measurements were taken from distinct samples or whether the same sample was measured repeatedly
☐	☒	The statistical test(s) used AND whether they are one- or two-sided<br>*Only common tests should be described solely by name; describe more complex techniques in the Methods section.*
☐	☒	A description of all covariates tested
☐	☒	A description of any assumptions or corrections, such as tests of normality and adjustment for multiple comparisons
☐	☒	A full description of the statistical parameters including central tendency (e.g. means) or other basic estimates (e.g. regression coefficient) AND variation (e.g. standard deviation) or associated estimates of uncertainty (e.g. confidence intervals)
☐	☒	For null hypothesis testing, the test statistic (e.g. F, t, r) with confidence intervals, effect sizes, degrees of freedom and P value noted<br>*Give P values as exact values whenever suitable.*
☒	☐	For Bayesian analysis, information on the choice of priors and Markov chain Monte Carlo settings
☒	☐	For hierarchical and complex designs, identification of the appropriate level for tests and full reporting of outcomes
☒	☐	Estimates of effect sizes (e.g. Cohen's d, Pearson's r), indicating how they were calculated

Our web collection on statistics for biologists contains articles on many of the points above.

Software and code

Policy information about availability of computer code

Data collection	No software was used
Data analysis	No software was used

For manuscripts utilizing custom algorithms or software that are central to the research but not yet described in published literature, software must be made available to editors/reviewers. We strongly encourage code deposition in a community repository (e.g. GitHub). See the Nature Research guidelines for submitting code & software for further information.

Data

Policy information about availability of data

All manuscripts must include a data availability statement. This statement should provide the following information, where applicable:

- Accession codes, unique identifiers, or web links for publicly available datasets
- A list of figures that have associated raw data
- A description of any restrictions on data availability

Our microarray data set of NQO1 knockdown and non-targeted control LNCaP cells (GSE58336) and RNA sequencing data from CTCs (GSE115501) are available in Gene Expression Omnibus (GEO) (https://www.ncbi.nlm.nih.gov/geo/). NQO1 expression in patient samples from multiple cohorts referenced in the study are available in Oncomine (http://www.oncomine.org/). The TCGA and SU2C/PCF data are publicly available (http://www.cbioportal.org/). Kaplan-Meier analysis of two cohorts (GSE40272; GSE70769) are available in PROGgeneV2 (http://www.compbio.iupui.edu/proggene). All other data supporting the findings of this study are available within the article and its supplementary information files. Raw data is provided.

Field-specific reporting

Please select the one below that is the best fit for your research. If you are not sure, read the appropriate sections before making your selection.

☒ Life sciences ☐ Behavioural & social sciences ☐ Ecological, evolutionary & environmental sciences

For a reference copy of the document with all sections, see nature.com/documents/nr-reporting-summary-flat.pdf

Life sciences study design

All studies must disclose on these points even when the disclosure is negative.

Sample size	Exact sample sizes are mentioned in all data. No sample size calculation was done in publicly available data sets. For the in vivo mouse and in vitro assays, sample sizes were chosen based on our experience and previously published literature.
Data exclusions	1. Animal studies: One animal in the shNQ group died from non-tumor related cause which was excluded from rest of the analysis (Fig. 1a).<br>2. Human TMA tissue: Out of a total of 26 BCR patients, 23 BCR patients were subjected to cumulative NQO1 staining analysis. 3 cases were excluded from analysis in Fig. 2f because adjacent normal prostate tissues were not available.
Replication	Experiments were repeated to confirm reproducibility of all assays. Appropriate markers and internal controls (negative, positive or loading as applicable) were used.
Randomization	Mice were ear tagged and randomly assigned to receive sh-NTC or sh-NQO1 cells in the tumor study.
Blinding	HM provided a set of human TMA (n=150) and following NQO1 staining (RGB), scoring was conducted in a blinded manner. For H&E staining of the embedded mouse prostate tissue, histopathological evaluation was conducted by a blinded pathologist (RLR).

Reporting for specific materials, systems and methods

We require information from authors about some types of materials, experimental systems and methods used in many studies. Here, indicate whether each material, system or method listed is relevant to your study. If you are not sure if a list item applies to your research, read the appropriate section before selecting a response.

Materials & experimental systems

n/a	Involved in the study	
☐	☒	Antibodies
☐	☒	Eukaryotic cell lines
☒	☐	Palaeontology
☐	☒	Animals and other organisms
☐	☒	Human research participants
☒	☐	Clinical data

Methods

n/a	Involved in the study	
☒	☐	ChIP-seq
☒	☐	Flow cytometry
☒	☐	MRI-based neuroimaging

Antibodies

Antibodies used

1. Immunohistochemistry:
NQO1 (Sigma, HPA007308): dilution (1:80)

2. Western Blot:
NQO1 (Sigma, N5288): dilution (1:7500)
β-actin (Sigma, A5441): dilution (1:7500)
HDAC1 (Santa Cruz, sc-6298): dilution (1:500)
α-tubulin (Santa Cruz, sc-6928): dilution (1:1000)
AR (Santa Cruz, sc-816): dilution (1:1000)
PSA (Cell Signaling Technology, 2475): dilution (1:2000)
PTEN (Santa Cruz, sc-133197): dilution (1:500)
Fibronectin (Santa Cruz, sc-9068): dilution (1:500)
TCF8/ZEB1 (Cell Signaling Technology, 3396): dilution (1:1000)
E-cadherin (Cell Signaling Technology, 3195): dilution (1:2000)
N-cadherin (Cell Signaling Technology 13116: dilution (1:1000)
Vimentin (Cell Signaling Technology, 5741): dilution (1:2000)
β-catenin (Cell Signaling Technology, 8480): dilution (1:2000)
Smad3 (Cell Signaling Technology, 9523): dilution (1:2000)
Smad2 (Cell Signaling Technology, 5339): dilution (1:2000)
Smad2/3 (Cell Signaling Technology, 8685): dilution (1:2000)
Smad4 (Cell Signaling Technology, 38454): dilution (1:2000)

LaminB1 (Abcam, ab16048): dilution (1:7500)

3. Immunofluorescense
E-cadherin (Cell Signaling Technology, 3195): dilution (1:250)
N-cadherin (Cell Signaling Technology 13116: dilution (1:100)
Vimentin (Cell Signaling Technology, 5741): dilution (1:250)
NQO1 (Sigma, N5288): dilution (1:500)

4. Cytokine array
Capture antibodies in nitrocellulose membranes- Human Cytokine Array, Panel A (R&D Systems, # ARY005)

Validation

All antibodies used were from well established commercial vendors. Manufacturers provided technical data sheet along with references to previous publications. In our lab, NQO1 antibody was validated using various transient and stable knockdown cell lines.

Eukaryotic cell lines

Policy information about cell lines

Cell line source(s)

ARCaPE and ARCaPM (Novicure Biotechnology)
LNCaP and PC-3 (ATCC)

Authentication

ARCaPE (epithelial clone) and ARCaPM (mesenchymal clone) cells were used within 8 passages of thawing. These isogenic clonal cells were not authenticated but confirmed based on morphology, AR expression and EMT-associated genes as per information provided by the vendor.

LNCaP and PC-3 cells were purchased from ATCC and not authenticated but were confirmed by expression or loss of AR, PSA and PTEN using western blot (Supplementary Fig 8).

Mycoplasma contamination

Cell lines were not regularly tested for mycoplasma contamination.

Commonly misidentified lines
(See ICLAC register)

Cell lines used are not on ICLAC register as of November 18, 2019.

Animals and other organisms

Policy information about studies involving animals; ARRIVE guidelines recommended for reporting animal research

Laboratory animals

Male athymic nude mice that were 4-6 weeks of age were used.

Wild animals

Study did not use wild animals

Field-collected samples

Study did not involve field-collected samples.

Ethics oversight

The University of Texas Health Science Center at San Antonio IACUC approved the animal study.

Note that full information on the approval of the study protocol must also be provided in the manuscript.

Human research participants

Policy information about studies involving human research participants

Population characteristics

N/A

Recruitment

We did not recruit subjects for this study.

Ethics oversight

De-identified samples were obtained in the form of TMAs (collection of tissues for the TMAs was approved by the University of Rochester Medical Center IRB. Data analysis of de-identified CTCs was conducted (GSE115501). CTCs were collected upon approval from UTHSCSA IRB.

Note that full information on the approval of the study protocol must also be provided in the manuscript.
